# Presenting fabrics in digital environment: fashion designers’ perspectives on communicating tactile qualities of the fabrics

**DOI:** 10.1186/s40691-022-00328-2

**Published:** 2023-02-15

**Authors:** Shin Young Jang, Jisoo Ha

**Affiliations:** Department of Textiles, Merchandising and Fashion Design, Seoul National University, 1 Gwanak-Ro, Gwanak-Gu, Seoul, Republic of Korea

**Keywords:** Haptic information, Tactile perception, Textile experience, Digital textiles, Fabric presentation, Fashion designer

## Abstract

The ongoing COVID-19 pandemic has forced the fashion industry to digitalize the conventional work system. Fashion designers were required to work from home, and textile trade shows were held online. However, fabric suppliers were unable to present their fabrics in a manner that enabled their properties to be easily understood. Visual information, such as photographs, videos, and the brief explanations provided by fabric suppliers were insufficient for fashion designers to comprehend the fabric’s properties. Thus, this research aims to identify the critical information for fashion designers in their digital fabric search and to discover effective ways to present this information. The current state of online tactile information was analyzed by conducting content analysis on several online fabric retailers. Then, semi-structured one-on-one in-depth interviews with 25 fashion designers were conducted to identify the strengths and weaknesses of the existing types of visual and textual information. Results revealed the most effective ways to present fabrics online. Specific guidelines were established for photographing or writing each type of information. Finally, a conceptual framework for fabric tactile communication in digital environments was developed. This study can contribute to the improvement of a designer’s experience in online fabric markets and can be used as a fundamental guide on designer’s tactile perception of fabrics, which can support technologies, such as haptic devices and 3D clothing simulation software.

## Introduction

As physical distancing guidelines have been enacted worldwide because of the COVID-19 crisis, the demand for and dependence on business communication through contactless methods is sharply increasing. Much of the world moved online, which accelerated a digital transformation in the textile industry. As professional settings rapidly digitized, the traditional work system of the fashion industry collapsed. International and domestic travel was restricted and various large public and private events were banned. Textile fairs and trade shows were held in digital form. Unfortunately, the textile industry was not ready for those changes. Companies were unable to clearly present their fabrics so that their properties could be understood.

It was especially hard for fashion designers to make any design decisions based on the information provided online. Visual information; such as photos, videos, and the explanation presented in a few words by online fabric suppliers, were insufficient for fashion designers to understand the characteristics of the fabric. For these reasons, the methods of fabric presentation in digital forms needs to be improved.

Unlike the textile industry, the apparel industry has continuously developed its presentation methods for decades to resolve consumer uncertainty. It tries to give as much specific information as possible by providing various photos, magnification functions, videos, and real-time chat services. In addition to these method for providing information, research (Chen et al., 2015; Volino et al., 2006) on haptic devices and additional services has been actively conducted to resolve the consumer's uncertainty of identifying the clothing item. However, there is still no method introduced by either industry that conveys a feeling similar to touching a real fabric. There is a noticeable gap between technology and real life. The mechanically measured and delivered tactile properties are not equivalent to the tactile perceptions of human hands touching the fabric (Chen et al., 2015). Therefore, a fundamental understanding of human tactile perception is necessary prior to the development of the haptic technologies (Chen et al., 2015).

However, most previous research in related topics focus on general consumers’ point of view, not designers. The way general consumers and fashion designers perceive fabrics is not the same (Petreca et al. 2015). Therefore, information that fashion designers search for when they shop for fabrics cannot be identified through the previous studies on general consumers. There is also a lack of academic research to provide specific guidelines for how companies should present fabrics online, and it is not clear what factors are important when showing fabrics digitally. Therefore, this study explores how fashion designers perceive fabrics online and discovers effective methods to present fabrics in digital environments. This study also aims to develop a conceptual framework for the tactile communication between fashion designers and the textiles industry in a digital environment. This study was designed with three research objectives. First, identify and analyze the current state of tactile online content. Second, uncover factors that influence how fashion designers perceive fabrics online. Third, determine an efficient method of presenting fabrics online to fashion designers.

We conducted content analysis on multiple online fabric stores to analyze the current state of tactile content online. Then, we conducted semi-structured one-on-one in-depth interviews with fashion designers to determine the strengths and weaknesses of the existing types of visual and textual information. The results revealed effective methods for presenting fabrics online, which were used to establish guidelines for photographing or writing the information for each type. Last, we developed a conceptual framework for fabric tactile communication in digital environments. The findings of this study will help to improve a designer’s experience in online fabric markets and can be used as a basic guide on a designer’s tactile perception of fabrics, which can support technologies, such as haptic devices and 3D clothing simulation software.

## Literature review

According to Xue et al. (2013), the majority of tactile fabric properties can be perceived visually, without touch. When humans see and touch an object, their brains store the tactile information gained from the multisensory experience. After the initial tactile encounter, visual representations of the object may evoke sensory memories (Jang & Ha, 2021). Thus, visual information can sufficiently deliver tactile properties when touch is deprived, but its accuracy depends on the level of information provided. When humans perceive information through a computer, their vision collects visual and tactile information through the website’s interface. Prior studies (Chiang & Dholakia, 2003; Hassanein & Head, 2007) proved that the amount of information, the way information is presented, and the design of the website have a significant impact on consumer behavior and the reliability of the website and products.

Numerous studies have been conducted to determine the relationship between websites and user satisfaction, and these studies have highlighted the significance of information quality (Bailey & Pearson, 1983; Delone & McLean, 2004; Negash et al., 2003). The quality of information can be determined by a single attribute, such as accuracy, but other attributes, such as understandability, reliability, and usefulness, affect the quality depending on the type and purpose of use.

A slightly different set of criteria for judging the quality of information is defined depending on the timeline. Accuracy, reliability, timeliness, relevancy, and confidence in the system are the most important factors (Bailey & Pearson, 1983). Later, the e-commerce perspective was added to the traditional criteria. Negash et al. (2003) saw two aspects of information quality: informativeness and entertainment. Accuracy, relevance, timeliness, ease of use, and comprehensiveness are elements of informativeness. Entertainment entails whether or not the interface is entertaining, pleasurable, enjoyable, fun, and exciting. This research indicates that the total experience of information quality, system quality, and service quality correlate with user satisfaction.

## Methods

### Participants

For this study, a total of 25 womenswear designers between the ages of 30 and 45 were selected as participants. Similar qualitative studies (Fernandes & Albuquerque, 2008; Baumgartner et al., 2013; Kahrimanovic et al., 2011) included 16 to 25 participants. Our goal was to have at least 20 people and no more than 30. In the initial stage, 27 people were recruited, whereas 25 people fully participated. According to a prior study (Musa et al., 2019), women tend to have better sensitivity in touch than men. Therefore, only female participants were recruited. The main criteria to select participants were a high tactile sensitivity and familiarity with apparel fabrics. In the fashion industry, associate designers with three years of experience are typically referred to as “experienced designers.” Therefore, an assistant designer level (1–2 years of experience) was excluded. All participants had over three years of fashion or textile design experience. The possibility of a distinction between fashion textile designers and apparel designers was anticipated. However, whether a difference exists between them has not been proven academically in this regard. Thus, this study was conducted with broad participants. Due to the paucity of prior research on this specific topic, this study aimed to elicit various opinions and uncover diverse points of view on the basis of the qualitative method. Participants were recruited through online message boards of womenswear fashion companies. Female fashion designers with over three years of experience were encouraged to participate. Given that fashion textile designers also consider themselves fashion designers, some of them were textile designers. Eight people were fashion (apparel) textile designers, and 17 people were apparel designers. Table 1 provides specific information about the participants. This research is approved from Seoul National University IRB for the interviews. The approval number is IRB No. 2108/001-004.

**Table 1:** Participants’ information

Number	Name(initial)	Age	Experience(year)	Occupation
1	JS	44	21	Fashion textile designer
2	WL	34	8	Fashion textile designer
3	KR	34	6	Apparel designer
4	YS	41	15	Fashion textile designer
5	AK	33	9	Apparel designer
6	HJ	41	17	Apparel designer
7	HS	32	6	Fashion textile designer
8	YJ	31	5	Apparel designer
9	NL	31	4	Apparel designer
10	BY	36	10	Apparel designer
11	JY	44	20	Fashion textile designer
12	SE	32	6	Fashion textile designer
13	CL	30	6	Apparel designer
14	EY	31	4	Apparel designer
15	YB	36	9	Apparel designer
16	HS	31	7	Apparel designer
17	SM	31	5	Fashion textile designer
18	KW	42	19	Apparel designer
19	SJ	38	13	Fashion textile designer
20	JH	29	4	Apparel designer
21	KN	29	4	Apparel designer
22	JS	30	4	Apparel designer
23	LK	33	7	Apparel designer
24	HE	35	8	Apparel designer
25	EH	31	5	Apparel designer

### Research process

This study used a qualitative research method. Qualitative research is used to identify various points of view on a phenomenon or problem and to identify related factors. It is effective for identifying the interaction of complex factors (Creswell, 2021). In this exploratory research, contents analysis on online fabric stores and in-depth interviews with fashion designers were conducted.

**Fig. 1:**
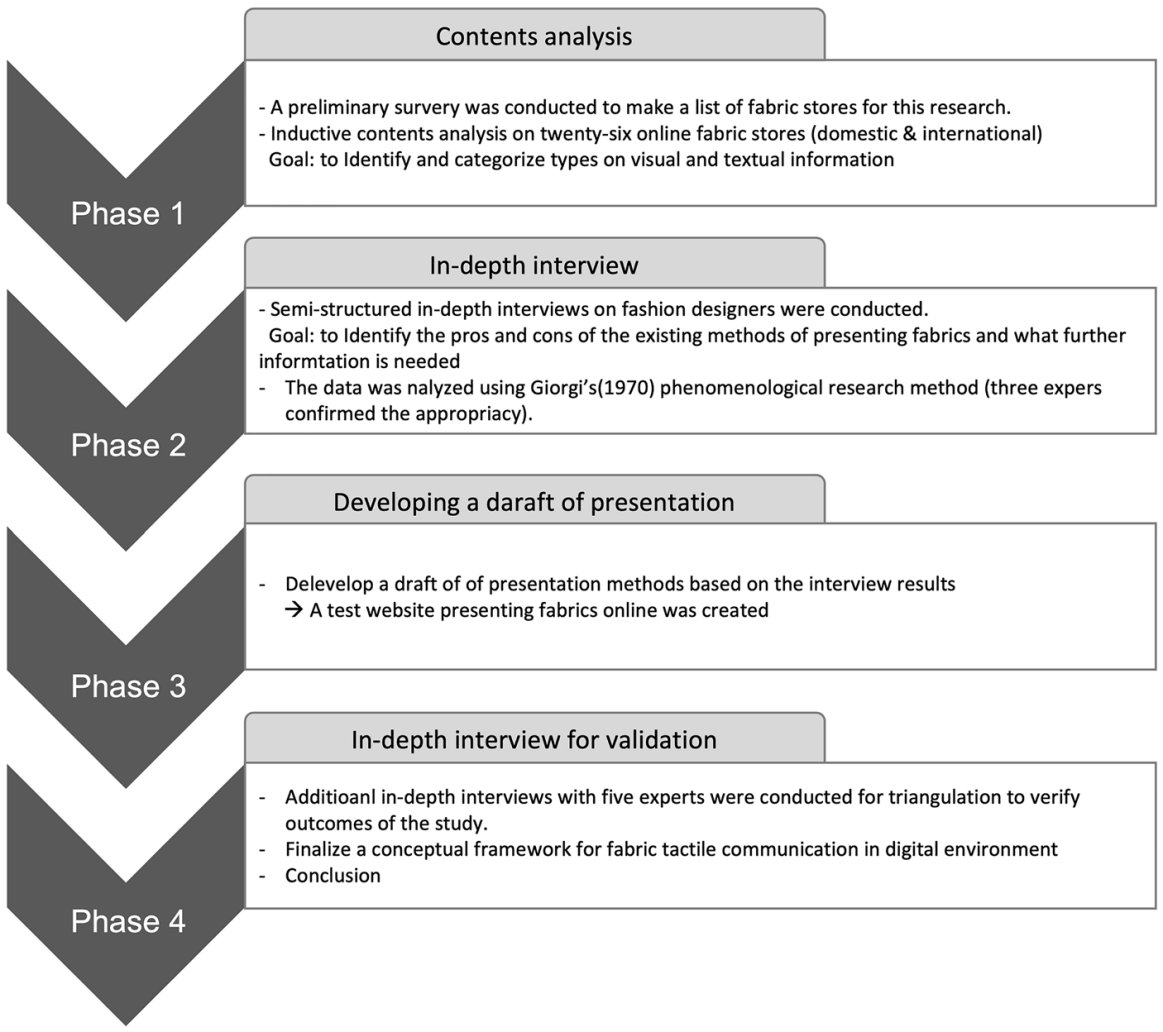
Research process

This research was processed in five steps (Fig. 1). First, inductive contents analysis on multiple online fabric stores were conducted to analyze the current state of tactile information provided online. Domestic (Korean) and international online fabric stores were analyzed to identify and categorize the types of visual and textual information currently used to describe fabrics online. A preliminary survey was conducted on twenty designers and fashion students who live in major fashion cities (New York, Seoul, Paris, London, Milan). They were recruited through a snowball sampling method. The preliminary survey included the following four questions.

1. Where do you usually shop for fabrics in the city? List names of the stores.
2. Do you shop fabrics online? If you have purchased fabrics online (even for personal use), please list the names of the website.
3. Even if you have never purchased fabric online, if you know of a website that sells fabric online, please let us know.
4. In a certain situation (e.g., COVID-19), if you have to shop fabrics online, where would you shop? How do you find websites?

Their responses to the first and second questions indicated that the majority of them shop offline. Moreover, their answers indicated that although there were numerous fabric companies, only few stores sell various types of fabric. The list of offline fabric stores they provided was searched to confirm if they have an online website. The search found two American and two British websites selling fabrics actively online and offline. From the second question, we found one Italian and two Korean websites. From the first two questions, seven websites were identified. No new websites were found from the third question, so more websites were added on the basis of the answer to the fourth question. Most of the participants (n = 16) answered that they would use search engine, such as Google, using the keywords “fashion fabrics online,” “online fabric store,” and verify the results once more. Implementing the same method as they suggested, more websites were found and categorized by their origin. On the basis of the results, 26 online fabric stores were selected: 10 American, 10 Korean, 2 British, 1 Italian, and 2 French. We set a maximum of 10 sites per country. The number of websites analyzed in each country was not equal because only few online fabric stores existed in European countries, in comparison to Korea and the United States. A broad range of fabrics, including cotton, polyester, silk, linen, wool, lace, jersey, and printed fabric were investigated on each website to confirm if there was a difference in their presentation.

Second, a semi-structured one on one in-depth interviews with fashion designers were conducted to identify the strengths and weaknesses of the existing types of visual and textual information. Based on the results from the contents analysis, researchers compiled a list of the types of information currently provided online, with examples. The list was then shown to the interview participants. And then, they reviewed it and shared their opinions and thoughts on it. The goal of these interviews was to identify what further information is needed to improve the accuracy of digital fabric recognition. Each interview was recorded and transcribed, and the transcripts were analyzed using Giorgi's (1970) phenomenological research method. Giorgi's method is used to elucidate the meaning of human experiences through transcriptions of in-depth interview. The data analysis method is divided into four steps (Giorgi & Giorgi, 2003). First, researchers must read and understand the content. Second, researchers discriminate meaning units. The third stage involves collecting meaning units together to create a meaning structure. In this stage, researchers transform the language of the participants into an academic expression. The themes and focal meaning of the participant’s interview are also identified at this time. Finally, the focal meaning is integrated to create a structural description of the participants’ perspective, and the general structural description, which represents the point of view of the entire participants, is made. Three experts having a PhD in clothing and textiles confirmed the appropriacy of the overall process and semantic units classified by the researchers.

Through the contents analysis and in-depth interviews, the first draft of the proposed method of online fabric presentation was developed. It went through a verification and supplement process with additional testing and interviews. A test page resembling a website for selling fabrics was created, and five industry professionals reviewed the online fabric presentation. The five experts have Ph.D. in fashion design and have worked for over 10 years in the fashion industry. The test page displayed nine fabric samples (Table 2), and the five experts were permitted to click and zoom in or out and were given unrestricted time to explore the page's content. After the exploration, each of them was interviewed for roughly 90 min. Finally, a conceptual framework for fabric tactile communication in digital environment was developed.

**Table 2:** Description of the sample fabrics

	Composition	Thickness (mm)	Stretchiness	Drape coefficient (%)
Cotton	Cotton 100%	0.25	warp: 2.7%, weft: 3.2%	66
	Cotton 100%	0.28	warp: 2.2%, weft 12.7%	46.9
	Cotton 95%, Polyurethane 5%	0.7	warp: 4.7%, weft: 22.7%	55.8
Polyester	Polyester 100%	0.24	warp: 7.5%, weft: 9.3%	42.3
	Polyester 100%	0.28	warp: 2.7%, weft: 16.7%	40.5
	Polyester 97%, Spandex 3%	0.6	warp: 3.7%, weft: 23.7%	67.8
Silk	Silk 95%, Lycra 5%	0.19	warp: 2.7%, weft: 54.5%	60.3
	Silk 100%	0.23	warp: 2.7%, weft: 3.7%	72.2
	Silk 95%, Lycra 5%	0.31	warp: 3.3%, weft: 35.2%	67.7

### Research scope

Our research focused on womenswear designers (ready-to-wear). A preliminary investigation was conducted to identify fabric types that are most challenging to identify in a digital setting. Twenty-eight womenswear designers with over three years of experience were surveyed. The survey comprised two simple questions on fabric types and tactile properties, and no visual information was provided to avoid any confusion. Our objective was to elicit spontaneous responses from their minds on the basis of their prior experiences. The result showed that a solid cotton, polyester, and silk is the hardest fabric types to “feel the touch” online. Four tactile properties; fabric thickness, stretchiness, luster, and drape, were identified as the most difficult characteristics to “feel” online. Therefore, this study focused on how information about thickness, stretchiness, luster, and drape is delivered when presenting cotton, polyester, and silk online. For each type, three varieties of fabrics with different thickness, luster, stretchiness, and drape properties were selected to create examples of visual information for the in-depth interviews (Table 2). After categorizing types of visual information based on the results from contents analysis, researchers made various sample photos and videos of each fabric type using the sample fabrics to refine the presentation method. A total of nine fabrics were selected, and the general characteristics of the fabrics were tested by KOTITI Testing & Research Institute (Table 2). Because a previous study by Xiao (2016) proved that color affects tactile perception of fabric, only black fabrics were used.

## Results

### Visual information

Twenty-five types of visual information were discovered from the contents analysis (Table 3). Most websites had a basic frontal photo of the fabric for sale and another photo of close-up texture. Some of them provided a high quality zoom-in function on the basic frontal photo instead of providing a second close-up photo of texture. In addition to the frontal and close-up photos, five types of photos showing folds on the fabric were identified: spiral-shaped creases, irregular creases, straight folds, folds using a hand (a hand is pictured in the photo folding the fabric), folds using a clothespin (to hold the folds like pleats). Spiral-shaped creases and irregular crease were most commonly used. Since these basic photos do not sufficiently illustrate the various fabric properties, some of the websites provided additional images.

**Table 3:** Types of visual information and overall perspectives of participants (the preferred methods are highlighted in red)

Type	Description	Transmittable tactile properties	Drawback
Front	Frontal photo	Texture, print size,	- Looks different depending on the shooting distance
	Close-up photo of the texture	Surface texture	- Looks different depending on the shooting distance and angle
Folds	Spiral-shaped creases	Flexibility, softness, thickness	- Difficulty in making the same spiral-shaped creases
	Irregular creases	Flexibility, softness, thickness	- Difficulty in making the same shape and the amount of the creases
	Straight folds	Softness, thickness	- Difficulty in making the same shape and the amount of the creases
	Folds using hand	Flexibility, softness, thickness	- Limited information can be obtained because some area is hidden in the hands
	Folds using a clothespin	Flexibility, softness, thickness	- Fabric may look different depending on how you use the clothespin
Cut edge	Folded edge	Thickness	- Limited information available compared to other types - In the case of a thin and flexible fabric, creating a standing fold is impossible
	Flat edge	Texture	- Limited information available compared to other types
	Role of fabric	Texture, thickness	- Limited information available compared to other types
	Fabric edge with a hand	Thickness, transparency	- Limited information available compared to other types
Transparency (Sheer fabric only)	Using a hand	Thickness, transparency	- Unable to check the exact level of transparency because it varies by lighting and hand movement
	Using a background color	Thickness, transparency	- May look different depending on the background color
Drape	Hanging on the wall	Drape	- Limited information available compared to other types
	Fabric on a dress form (photo)	Drape, flexibility, softness, thickness	- May look different depending on how the fabric is placed
	fabric on a dress form (video)	Drape, flexibility, softness, thickness	- Difficult to observe the natural appearance of the fabric
	Fabric on a object (E.g. basket, chair)	Drape, flexibility, softness, thickness	- Distracting and unprofessional
	Draping on a dress form	Drape, flexibility, softness, thickness	- The style (design) of draping can affect the judgment on the use of fabric and aesthetic value
	Using a salesperson and dress form	Drape, flexibility, softness, thickness	- Difficult to focus on the fabric itself - The duration of video is lengthy - The subjective explanation of the salesperson can adversely affect the objective judgment of the fabric
Stretchiness	Using hands to stretch (photo)	Stretchiness	- Impossible to feel the exact elasticity through the still image
	Using hands to stretch the fabric (video, gif)	Stretchiness	- Difficult to confirm how much force is applied to stretch the fabric on the video
Waterproofing property	Using water (photo)	Surface finishing	- Difficult to distinguish between waterproof and water repellent
	Using water (video, gif)	Surface finishing	- Difficult to distinguish between waterproof and water repellent
Worn by a model	Wearing a production sample (photo)	Drape, flexibility, softness, thickness	- The type of the item and styles of the garment can affect objective judgment on the fabric
	Wearing a production sample (video, gif)	Drape, flexibility, softness, thickness	- May look different depending on the size of the model and the model’s movements in the video - The type of the item and styles of the garment can affect objective judgment on the fabric

**Fig. 2:**
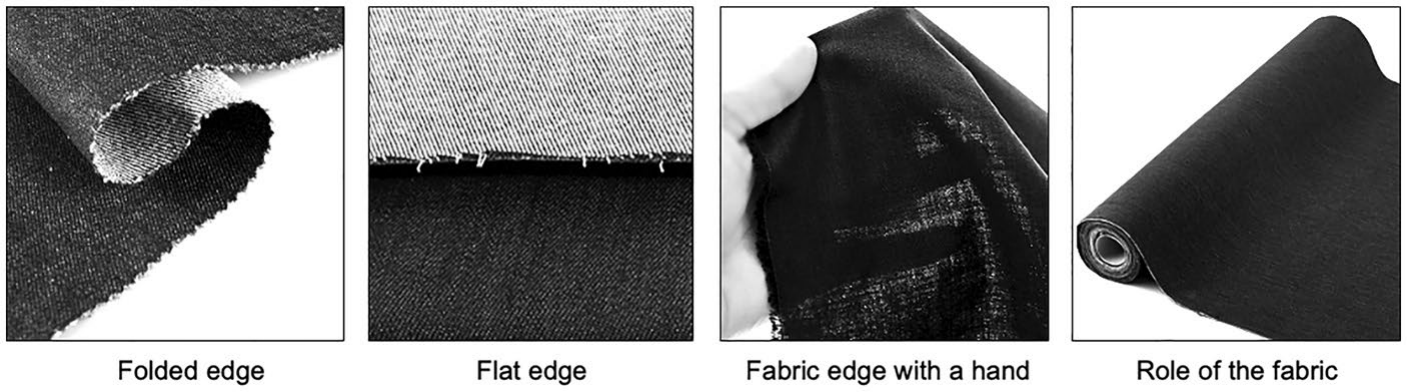
Various methods of showing the fabric edges

As supplements, four types of photos showing the fabric edges were identified (Fig. 2): folded edge, flat edge, fabric edge with a hand, role of the fabric.

For sheer fabrics, two types of photos showing the transparency of fabric were found: using a hand, and using a background color. Also, there were four photos and two videos showing how the fabric drape. The photos showed the fabric hanging on the wall, on a dress form, on an object such as a chair and basket, and draping on a dress form. One video showed the fabric on a dress form and the other used both a salesperson and dress form. Most of the websites did not provide visual information about stretchiness, but a few of them had a photo or video that used hands to stretch the fabric. For waterproof fabrics, some of the websites provided a photo or video of water pouring on the fabric to show its waterproofing property. Videos or photos of a model wearing a production sample using the fabric was also found.

In analyzing these types of visual information, fashion designers were interviewed to confirm or reject the effectiveness of each type. While all participants agreed that five to ten forms of visual information are a proper amount to present, the eight most favored (n ≥ 18) forms of visual information included six photos and two videos. The six types of selected photos were as followed: a frontal photo, a close-up photo of the texture, a photo of spiral-shaped creases, a photo of irregular folds, a photo of the fabric on a dress form, and a photo of draping. Besides these photos, two videos were selected: a video showing the stretchiness of the fabric, and another video of fabric moving with a hand. Although all participants agreed that a video showing stretchiness is essential, they desired a different method for presenting the stretchiness, as the current presentation method was not effective.

**Fig. 3:**
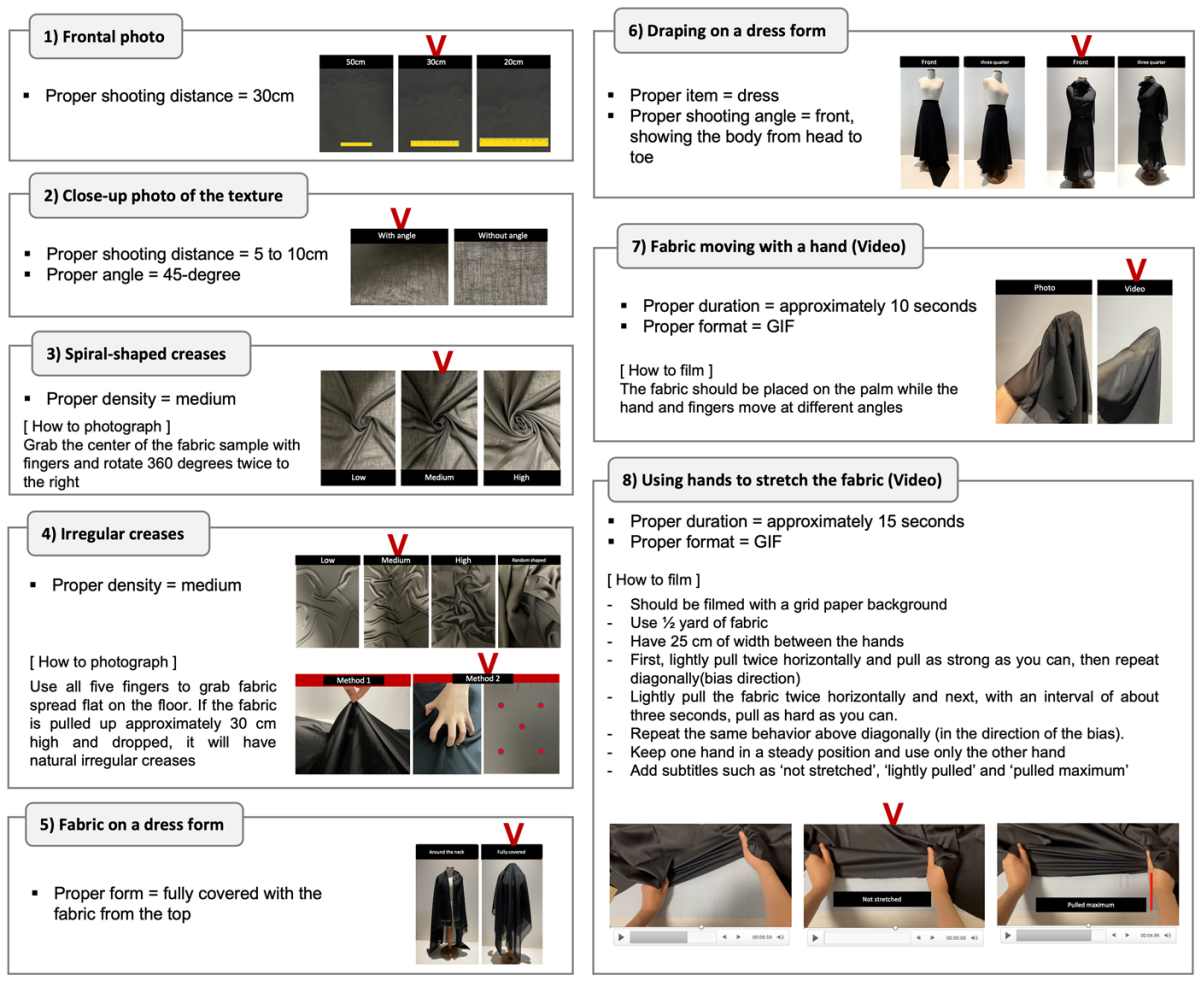
Refinement of the visual presentation method and guidelines for filming

Given that numerous methods exist for describing each type, testing and a second interview with fashion designers were required to determine the optimal presentation method. Five experts with a Ph.D. in fashion design and over 10 years of experience as fashion designers were interviewed. On the basis of the results of the earlier interviews, alternatives were developed for each type. The options for each type were presented and discussed with the experts. To create a test page, methods on which more than half of the experts agreed were chosen from the available alternatives. Finally, they reviewed a test page displaying fabrics that resembled websites. Through these additional in-depth interviews, each type's presentation method was refined and confirmed (Fig. 3). For example, a frontal photo, which is the most basic form of visual information in this industry, should be presented with a ruler placed on the top of the fabric and taken at a distance of 30 cm from the fabric. If the distance is too close, the surface texture is exaggerated. By including a ruler on the fabric, designers can approximate how close the photo is taken and they can see the pattern size if the fabric is printed. To best deliver the fabric’s texture, a close-up photo should be taken within a 5 to 10 cm distance and at a 45-degree angle.

For photos of a spiral-shaped crease and irregular folds, medium density was most preferred. The term ‘medium’ is best described in the photo below (Fig. 3). To create a medium density spiral-shape, one grabs the center of the fabric sample with their fingers and rotates 360 degrees twice to the right. To photograph irregular creases, one uses all five fingers to grab fabric spread flat on the floor. If the fabric is pulled up approximately 30 cm high and dropped, it will have natural irregular creases.

For a photo of fabric on a dress form, the dress form should be fully covered with the fabric from the top to show the drape and transparency of the fabric. In a photo of draping, designers agreed that a simple dress shape is great for all fabric types and the photo should be taken from the front, showing the body from head to toe.

For the video of fabric moving with a hand, the fabric should be placed on the palm while the hand and fingers move at different angles. The proper duration is approximately 10 s. This video shows not only how the fabric drapes, but also various other properties such as thickness, transparency, luster, flexibility, color, and texture.

To show stretchiness of fabric, the video form using hands to stretch the fabric should be filmed with a grid paper background to see changes in the length when the fabric stretches. It is recommended to use one half yard(45 cm) of fabric.

…If the fabric sample is too small, it is hard to see how stretchy it is even when videos are provided. If you grab the usual swatch size (less than10cm x 10cm) and stretch it, I cannot see how the fabric moves well because of your fingers. I think I can catch other characteristics of the fabrics, like the structure, too, if the fabric sample is big enough to observe. (Participant 17, personal communication, September 14, 2021)

For filming, having about 25 cm of width between the hands is recommended. To film, one should lightly pull the fabric twice horizontally and next, with an interval of about three seconds, pull as hard as they can. Then, repeat the same behavior diagonally (in the direction of the bias). During the filming, keep one hand in a steady position and use only the other hand, so viewers can better understand how much the fabric can be stretched. Also, participants agreed that although it is a subjective expression, a video with subtitles such as ‘not stretched,’ ‘lightly pulled,’ and ‘pulled maximum’ is very helpful. The proper duration of this video is approximately 15 s with a maximum of 20 s. For all videos, a GIF format that does not require clicking a button to play was preferred. All of the above guidelines for visual presentation methods were created based on the interview results with the experts.

### Textual information

Through the contents analysis, five methods of textual information were identified: descriptive, fact-listing, sensory scale, icons, and own standard (Table 4).

**Table 4:** Types of textual information and overall perspectives of participants (the preferred methods are highlighted in red)

Type	Description	Advantages	Disadvantages
Descriptive	- Description of the fabric’s characteristics with three or more sentences	- Feels kind and familiar, easy to understand - Can explain details that are hard to present in other types	- Poor legibility due to the lengthy sentences and writing style - Use of subjective expressions decreases the credibility of the information - Sounds unprofessional
Fact-listing	- Presenting objective information briefly with a few words or numerical values	- Can deliver substantial objective information quickly - Feels professional and it is easy to read	- Converting numerical information into senses can be difficult
Sensory scale	- Presenting tactile characteristics on three- or five-point Likert scales	- Visually organized - Helpful as a supplementary material	- Low credibility because it is considered a subjective opinion - Can be perceived differently depending on word choices
Icons	- Used to deliver certain fabric characteristics	- Can be recognized quickly	- Limited information available compared to other types
Own standard	- A seller building a standard within their website	- Easy to compare with other fabrics within the same website	- Low credibility as the standard is created by the website

First, the descriptive type refers to the use of a description of the fabric’s characteristics with three or more sentences. This type of information sharing was not preferred because it is subjective and does not sound professional. One fashion textile designer with 15 years of experience said,

…When the description says the fabric is ‘thick,’ it is a personal opinion. I do not always think the fabric is thick according to my standards, because I can assume its thickness by checking the fabric weight in numbers. When my opinion and the description does not match, I feel frustrated. (Participant 4, personal communication, September 12, 2021)

Most fabric descriptions contain expressions such as 'soft,' or 'stiff,’ but these features are determined by subjective evaluation and should not be presented in descriptive writing.

Second, the fact-listing type presents objective information briefly with a few words or numerical values. All participants shared that when information is presented in this format, it feels professional and it is easy to read quickly. It was found that when information is presented through the fact-listing type, there is a tendency to not feel overwhelmed, even if the amount of information is copious. Participants with high level of experience (more than ten years) emphasized that they do not want a large amount of information when viewing a fabric because it takes time to read it all to find only the essential information. This conclusion suggests that the amount of information given is perceived differently depending on the type of information. Therefore, selecting an appropriate presentation method is a significant step in effective tactile communication.

Third, the sensory scale refers to the method of presenting tactile characteristics on three or five-point Likert scales. This type was only found on Korean websites, where the surface texture, thickness, transparency, luster, stretchiness, weight, and season of fabrics were presented in this format. During the interviews, there was a difference in opinions about this scale between apparel designers and textile designers. Almost all textile designers (n = 7) observed that the information presented in this type is too subjective to trust, so they do not need it. A textile designer with 18 years of experience said,

…I can imagine the actual fabric if I can see the composition, weight, and how the fabric is constructed, treated, and dyed. I think all textile designers can do the same. For me, the subjective evaluation on the sensory scale is not helpful at all; rather confusing. (Participant 19, personal communication, September 7, 2021)

However, most fashion designers (n = 15) mentioned that it is helpful, even if it is subjective information. An apparel designer with 19 years of experience said,

…It is hard to feel properties like stretchiness, surface texture, transparency, and luster online through visual information or detailed composition. For example, because the lighting affects visual information a lot, depending on the lights, it (fabric) could look opaque although it is actually sheer. In this kind of case, it is better to see the seller’s opinion, even though it is subjective. (Participant 18, personal communication, September 12, 2021)

According to the interviews, apparel designers want to acquire factual information in a fact-listing format, such as the fabric's composition, type, weight, dying method, and Pantone color number. Subjective information, such as stretchiness, surface texture, and luster, which is difficult to make objective in a textual format, was preferred to be viewed visually rather than through textual information. However, they noted that the delivery of visual information also has limitations, so a sensory scale should be utilized for supplementary purposes. Participants (n = 19) agreed that presenting information about thickness and seasonality in this method is not effective. Some apparel designers (n = 6) thought that showing thickness in the scale could be helpful.

Fourth, icons can be used to deliver certain fabric characteristics. For example, if the fabric is stretchy, a two-way arrow or four-way arrow symbol is presented on the website. Because all information cannot be presented in icons, this type of textual information limits the information that can be delivered. Almost all participants (n = 24) agreed that this type is unnecessary because it is difficult to present thorough details.

Fifth, the own standard refers to a seller building a standard within their website. Some websites have their own standards, and define the levels of thickness or stretchiness through terms such as 1T, 2T, 3T. Their criteria were further explained on the websites. All participants did not necessarily consider this type essential.

**Fig. 4:**
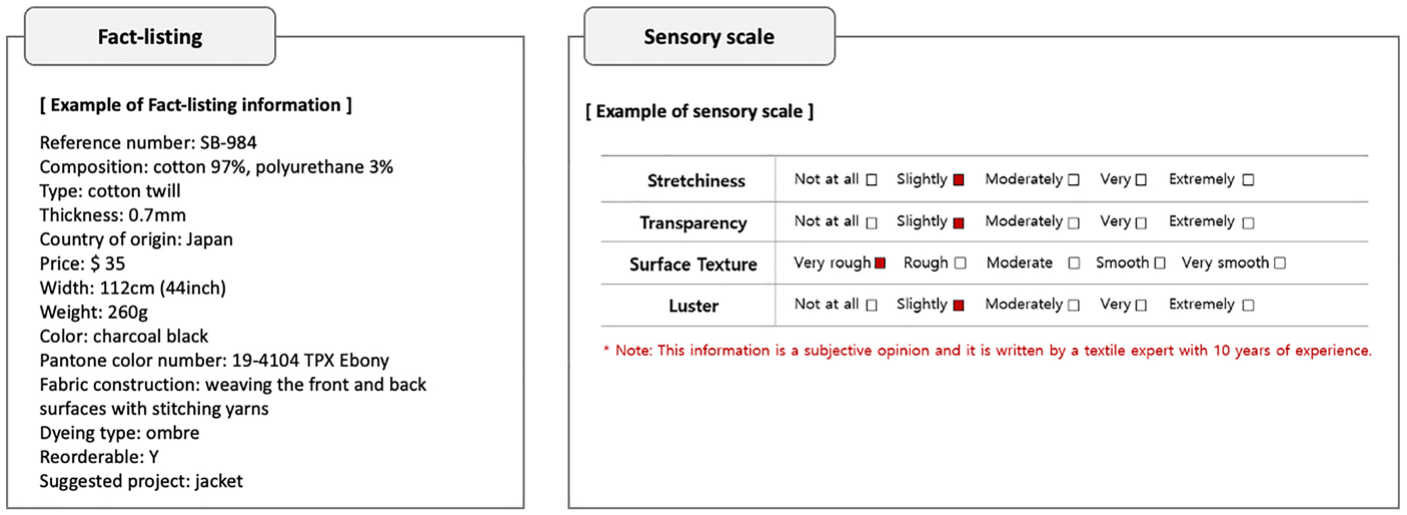
Suggested format of textual information

As a result, fact-listing type and sensory scale were identified as the most effective presentation methods to satisfy both fashion designers’ and textile designers’ needs (Fig. 4). Objective deliverable information should be presented with the fact-listing type: product number, composition, texture, thickness, country of origin, price, width, weight, color, Pantone color number, fabric constriction, fabric treatment, dyeing type, reorder availability, suggested project, and print size. However, most of the current websites provide only basic information, such as country of origin, price, width, color, and reorder availability. Interview participants, especially all textile designers, wanted to also know how the fabric is constructed, treated, and dyed. Participant (n = 20) agreed that Pantone color number is very helpful when they cannot see the actual fabric in person, because color looks very different depending on the screen setting.

On a sensory scale (Fig. 4), information on the fabric that is difficult to present with the fact-listing type, including stretchiness, transparency, surface texture, and luster, should be presented using a five-point scale. Most participants (n = 21) recommended to limit the amount of the sensory scale information to no more than five characteristics. Although participants (n = 16) agreed that this type of information is helpful, they expressed that its reliability is low because it is written based on subjective opinions. To increase its reliability, websites need to provide information about the writer so that the readers can ensure the information comes from an expert. An apparel designer with 9 years of experience said,

…If the website admits that the sensory scale is subjective and lets the users to know this, the website’s credibility increases. And the website should ensure its users that the rest of the information in different types is objective. When they try to deceive users to believing subjective opinions as objective facts, the credibility of the website decreases sharply. (Participant 5, personal communication, September 12, 2021)

All participants agreed that the information should be prepared by an expert in order to increase the reliability of the information and the professionalism of the website. Information on the experience of the expert who wrote the textual information should be presented. If it is stated on the website that the information presented through the sensory scale is a subjective opinion and the website also includes information of the writer, the overall reliability of the website can be improved. Also, because the fabric properties can be perceived differently depending on the words used in the scale, standardization of the expression across the website and industry is necessary. The appropriacy of the words used to describe fabric was verified and most of the participants (N = 23) agreed on the best descriptors in the example of the sensory scale (Fig. 4).

### Conceptual framework

**Fig. 5:**
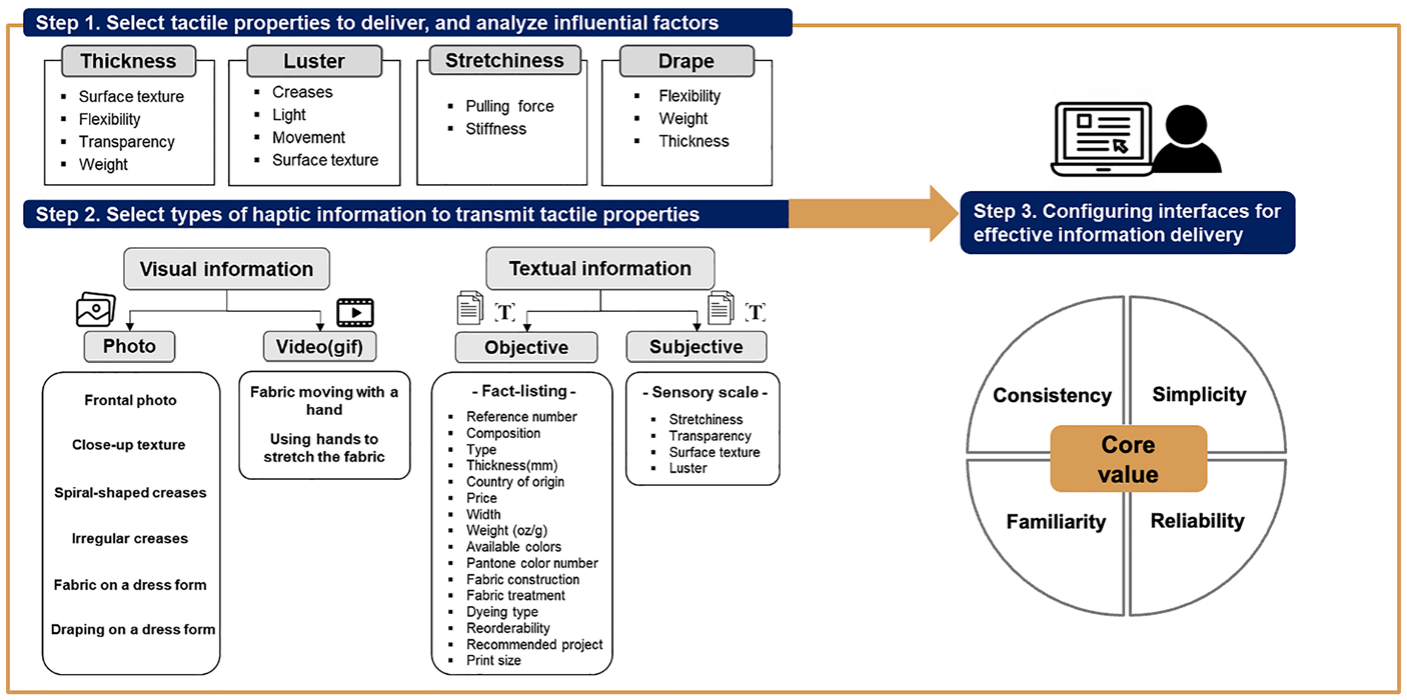
Conceptual framework for fabric presentation in digital environments

Based on the results above, a conceptual framework of online fabric presentation was developed (Fig. 5). To make an effective presentation, one must consider what tactile properties they want to deliver and then, they need to identify the influential factors. Because we limited the analyzed tactile properties to thickness, luster, stretchiness and drape in this study, the same four properties are presented in Fig. 5.

A prior study by Jang (2022) identified the most influential factors for the four properties. The study conducted experiments and interviews with fashion designers to identify how they perceive each property when they physically feel and touch the fabric in person. According to the study, designers perceive thickness of fabric based on its surface texture, flexibility, transparency, and weight. For luster, the researchers identified creases, movement, surface texture, and how the light reflects and absorbed as influential factors. For stretchiness, fabric stiffness and the pulling force of hands when they stretch the fabric influenced the designers’ perception. Last, it was proven that designers determine how the fabric drapes based on the fabric flexibility, weight, and thickness.

In consideration of the target tactile properties and their influential factors, specific types of visual and textual information were selected in this study as the best for proper digital perception of the fabric. For visual information, six photos were chosen, including the frontal photo, close-up photo of the texture, photos of spiral-shaped ceases and irregular creases, fabric on a dress form, and draping on a dress form. In addition, two videos were selected in this study: fabric moving with a hand and using hands to stretch the fabric.

For textual information, objective information should be presented with the fact-listing type and it must include the following information: reference number, composition, type, thickness, country of origin, price, width, available colors, Pantone color number, fabric construction, fabric treatment, dyeing type, reorder availability, recommended project, and print size. Subjective information such as stretchiness, transparency, surface texture, and luster should be provided through a five-point sensory scale.

In the end, this visual and textual information is presented on the web interfaces. Therefore, configuring these interfaces for effective information delivery is significant. The most important values are consistency, simplicity, familiarity, and reliability.

### Key factors for the effective presentation

#### Consistency

Participants mentioned words such as 'manual,' and emphasized the importance of consistency of the information presented online. This indicates the importance of the overall layout of the web interface and its content.

…When we shop apparel online, most of the popular websites are very well organized. I have never felt lost. But when I shop fabrics online, something is always not right. The format of information-giving and the way they describe fabrics is unsystematic. Most of them do not look professional at all. (Participant 21, personal communication, September 8, 2021)

All participants also agreed that when information about fabrics is presented unsystematically within the website, it not only takes a longer time to recognize the information, but the users also assume the website is not for professional use. This suggests that consistency is in connection with reliability. For a consistent user experience, all web pages must present information in the same format and follow the same rules.

Participants (n = 19) also stressed the importance of consistency with vocabulary used in the textual description. They mentioned that when the words used in the description are different within the same website, they cannot perceive the fabric well.

…I feel very confused by the expressions in descriptions. For example, if they have chosen to describe a heavy-weight fabric using a word ‘thick,’ all the heavy-weight fabrics on the website should be described using the same word. When they use different words randomly, like ‘for winter,’ ‘warm,’ ‘for F/W season,’ ‘heavy,’ or ‘thick’ to explain the same characteristics, it sounds very ambiguous. (Participant 6, personal communication, September 12, 2021)

These comments indicate that when retailers describe fabric properties, they need to build a manual for systematic characteristic descriptions and limit the use of ambiguous expressions. For professional use, designers agreed that if it is possible, presenting fabric in measured thickness(mm) or weight (g or oz) is more professional than describing it using adjectives.

#### Simplicity

Participants (n = 20) agreed that both quantitative descriptions and structural simplicity in visual and textual information is important for perceiving information online. They preferred to see essential information in a simple structure, because designers need to look at as many fabrics as possible within a limited business time.

…If I have to see a bunch of images of fabrics, like more than 10 images, I will be overwhelmed. Scrolling makes me tired. What we need is only the essential information, but I feel like retailers do not understand how and what we want to see. They are just giving us everything, throwing all the information (at us). This is not effective at all. (Participant 9, personal communication, September 11, 2021)

Participants emphasized the simplicity of the information by mentioning words such as 'short,' ‘simple,’ 'readable' 'concise,' ‘essential,’ and 'necessary.' Some of the participants (n = 12) also mentioned that simplicity is related to consistency.

…Even if you give the same amount of information, depending on how you organize and present it, the information could either feel overwhelming or easy to read quickly. If the explanation is long, the readability decreases and no one will use this kind of website for business. (Participant 9, personal communication, September 11, 2021)

This suggests that when the information is organized in a consistent way, users feel it is concise. Thus, presenting only the necessary parts in a short and concise form is recommended.

In terms of visual information, all participants agreed that a minimum of five to a maximum of 10 photos or videos are a proper amount. In comparison, however, they were less sensitive about the amount of textual information. Designers were open to as much information as possible, especially if the information is provided in fact-listing format, which has simple structure.

#### Familiarity

Ironically, although all participants were not satisfied by the existing format of online fabric presentation, a tendency amongst participants for a desire to see information in a familiar format was found. All participants mentioned that the presentation methods need to be developed. However, they were not fully open to websites using technologies, like haptic devices, unless they are proven to perfectly deliver the needed haptic information. It is especially true for business use, for which they need to see a lot of fabrics in a limited time, that they preferred to see things in easy and simple format. They felt the information is easy to read when the methods are familiar to them. An apparel designer with 19 years of experience said,

…I do not know well about the fancy technologies. Can we actually use it now? … Look at the online shopping websites for clothes now. When they first started selling clothes online, only few people used the service. But now everyone shops clothes online. I remember it has been developed step by step every year, starting from the very basic, ineffective website to now. Why should fabrics not be the same? (Participant 18, personal communication, September 10, 2021)

A textile designer with five years of experience said,

…Personally, I really wish there was a device that could perfectly deliver the tactile properties of fabrics and want to use it. However, as an employee at a company, we are a very conservative group. We follow traditional ways of seeing fabrics as my boss does. People usually hate when they have to change their work habit that has been continued for years. (Participant 17, personal communication, September 9, 2021)

This indicates that there is still a lot to be developed in the existing format of fabric presentation. Designers do not want to jump into a new technology that is not perfectly functioning. Therefore, the online presentation of fabric should be developed step by step, giving the users enough time to be familiar with it and adapt easily.

#### Reliability

Lastly, reliability was emphasized by the participants. All participants tend to find reliable information by distinguishing between the objectivity and subjectivity of the both visual and textual information. When they saw existing types of online fabric presentation, they naturally classified the objectivity or subjectivity of the types, even though no one asked them to do so.

In particular, they emphasized the reliability of textual information, which they agreed is the most important factor if they look for business use rather than personal use. When they see fabrics for business use, they tend to exclude subjective information when deciding which to purchase. When participants describe what they want on the websites, they commonly mentioned words such as ‘trustworthy,’ ‘objectivity,’ ‘reliable information,’ ‘reliable sources,’ and ‘professionalism.’ There was a tendency amongst designers that they do not trust written information on the website because they think that it could be written by a non-expert. Since they want to use the fabric for business purposes, if the information on the website seems inaccurate and not professionally described, the reliability on the website and the designer’s intention to use it website drops sharply. One participant spoke about the significance of a reliable source.

…When you describe fabric, you naturally evaluate the characteristics based on your judgement. I think it is okay, as long as the evaluator is a reliable expert. Honestly, if (website descriptions) stick to the objective information only, I think there is no way to describe fabric well. (Participant 8, personal communication, September 7, 2021)

In analyzing visual information, it was found that the reliability depends on the consistency of the presentation method. When the user feels that there is a difference in the filming method for each fabric, it can be perceived as deceitful information. In particular, in the case of creasing and draping photos, which are taken by manipulating the fabric, some participants mentioned that the reliability may be lower than that of the fabric taken without manipulation of the natural appearance of the fabric although they preferred to see those methods.

## Discussion

Some interesting insights were found from the participant interviews. Psychologists have said that our eyes tend to see as concisely as possible, and that conciseness translates to structural simplicity, not a quantitative conciseness (Chun, 2008). However, all participants emphasized both quantitative and structural simplicity in both visual and textual information. They preferred to see essential information in a simple structure, because designers need to check as much information as possible within a limited business time. In order to provide information concisely, using a layout that minimizes scrolling is recommended.

Also, individual experiences and background knowledge were also identified as major influencing factors on tactile perception of fabric in the digital environment. A difference between apparel designers and textile designers and their perception was found, even though they are all in fashion industry (Table 5).

**Table 5:** Differences between textile designer and apparel designers

Position	Level of Experience	Type of information		
		Perspectives on a photo of draping	Perspectives on textual information in numbers	Perspectives on textual information in sensory scale
Textile designer	High (above 6 years)	Neutral - Little influence on the style of the photo - Helpful but not necessary	Positive - Preferred to see weight or thickness in numbers - Can recognize the fabric and predict its properties accurately	Negative - Too subjective to trust, so they do not need it
	Low (3–5 years)	Neutral - Little influence on the style of the photo - Helpful but not necessary	Neutral - Can guess approximately but cannot recognize accurately	
Apparel designer	High (above 6 years)	Positive - The style of the photo affects judgment on the use of fabric - Helpful to see the possibilities of the fabric	Neutral - Can guess its weight approximately but cannot recognize the accurate feeling of touch	Positive - Helpful, even if it is subjective information
	Low (3–5 years)	Negative - The style of the photo affects judgment on the use of fabric and aesthetic value - Limits designer’s creativity and affect their design	Negative - Numbers cannot deliver an accurate feeling of touch	

Some of the apparel designers (n = 6) mentioned that visual information, like a photo of draping, can affect their judgment on the use of fabric and aesthetic value. Participants, especially those with a low level of experience, shared that this information can limit their creativity and affect their design because it already shows a certain style of apparel. However, apparel designers with higher level of experience preferred to see the photo as it is helpful to see the possibilities of the fabric. On the other hand, it was found that most textile designers (n = 7) received relatively little influence on the style of visual information.

With textual information, textile designers with many years of experience preferred to see weight or thickness in numbers, so they can recognize the fabric and predict its properties accurately. However, apparel designers and textile designers with low level of experience tend to think that numbers cannot deliver an accurate feeling of touch. Apparel designers with low level of experience shared that it is too hard to guess its properties by numbers although it is an objective way of presentation. Textile designer with low level of experience said that they can guess some properties by numbers but there is very low certainty. This shows that the ability to convert numerical information into senses can be different among the participants, depending on their experience levels. In sensory scale, textile designers tended to consider the information shown in the scale too subjective to trust, so they do not need it. However, most fashion designers found the information is helpful although they think it is subjective.

Unlike the offline environment, where one can feel the fabric by directly touching it, the digital environment requires visual and textual information and stored memory in brain work together to imagine and predict the touch. Therefore, the more experience one has with the type of fabric, the more accurately one can recognize the fabric well online. This suggests that in the future, a follow-up study should be conducted to clearly distinguish the differences between the apparel designer and textile designer group and to compare their cognitive differences, according to the level of experience by group.

## Conclusions

This study examined how fashion designers perceive tactile information about fabrics and how fabrics should be presented in order to be accurately recognized in a digital environment. Overall, the simplicity, consistency, familiarity, and reliability of information are key factors to delivering information effectively. This study examined the effect of existing presentation methods and then refined them. According to a prior study on human–computer-interaction by Oh (2006), delivering information in a way that is familiar to users maximizes communication effects. When information is transmitted to the brain through sight, information is organized into a state that can be easily remembered or characterized (Kok & de Jong, 1980). Therefore, the design of the website interface must be presented in a way that can be easily remembered and characterized. Therefore, the online presentation of fabric should be developed step by step, allowing enough time for the users to become familiar with it and adapt easily. The development of presentation should be based on how the designers physically feel fabrics in a traditional way, offline. Before jumping into the development of new technology, it is crucial to comprehend how designers feel about fabrics to develop an effective online presentation method. Therefore, additional research should also be conducted on this subject.

Although this study attempted to develop an effective presentation method, tactile recognition is about the subjective senses of humans and, therefore, it is difficult to create sufficient objective and reliable presentation methods used for business. The result of this study gives guidelines for filming and photographing proper media, but it is still difficult for textile retailers to capture consistent images because of multiple changing variables. Online fabric shops cannot always use existing methods, such as the Kawabata Evaluation System, to measure fabric properties because they require special devices and trained personnel to test. Thus, an accessible and objective method to measure fabric properties and deliver precise information to users in a digital environment must be developed further. A specific manual for capturing the media is important, because the consistency is conveyed by shooting in the same environment in the same way every time. It provides users with a consistent experience throughout the website and enhances the professionalism and dependability of the website, resulting in a positive user experience.

Despite some limitations, this study's findings contribute to a broader academic understanding of effective digital marketplaces in the fashion industry. As there are very few prior studies on the method of presenting tactile information of fashion fabrics in the digital environment, this study can serve as a foundation for future research. Here is the significance of this study: First, it can serve as a foundation for enhancing the service environment of 3D clothing simulation software such as 3D CLO, which requires fashion designers to select fabrics by predicting their tactile properties on-screen. Second, it aids in the expansion of the online fabric market. If online fabric stores and fabric fairs improve their services, more people will shop for fabrics online. Third, fashion designers can increase their productivity by decreasing the time required to collect fabric swatches in person.

In addition, the following environmental significance is attached to this study. Currently, when fashion designers collect fabric swatches, they typically do so without taking the resulting waste into account. If the fashion fabric market is successfully digitized, swatch requests can be made online with greater caution than offline, which will result in changes to consumer behavior. Online fabric markets will eventually contribute to sustainable practices by decreasing waste. In addition, the budget for manufacturing and distributing numerous swatches will be decreased.

## Data Availability

The datasets generated and/or analyzed during the current study are available from the corresponding author on reasonable request.

## References

[CR1] Bailey JE, Pearson SW (1983). Development of a tool for measuring and analyzing computer user satisfaction. Management Science.

[CR2] Baumgartner E, Wiebel CB, Gegenfurtner KR (2013). Visual and haptic representations of material properties. Multisensory Research.

[CR3] Chen S, Ge S, Tang W, Zhang J, Chen N (2015). Tactile perception of fabrics with an artificial finger compared to human sensing. Textile Research Journal.

[CR4] Chiang KP, Dholakia RR (2003). Factors driving consumer intention to shop online: An empirical investigation. Journal of Consumer Psychology.

[CR5] Chun, B. (2008). *Studies of efficient web interface design based on Gestalt’s law of visual perception* [Master's thesis, Ewha Woman’s University Graduate School of Design]. Ewha Research Repository. https://dspace.ewha.ac.kr/handle/2015.oak/176944

[CR6] Creswell JW (2021). A concise introduction to mixed methods research (second).

[CR7] DeLone WH, McLean ER (2004). Measuring e-commerce success: Applying the DeLone & McLean information systems success model. International Journal of Electronic Commerce.

[CR8] Fernandes A, Albuquerque PB (2008). Tactile perceptual dimensions: A study with light-weight wool fabrics. Haptics: Perception, Devices and Scenarios.

[CR9] Giorgi A (1970). Psychology as a human science: A phenomenologically based approach.

[CR10] Giorgi AP, Giorgi BM (2003). The descriptive phenomenological psychological method. Qualitative Research in Psychology: Expanding Perspectives in Methodology and Design..

[CR11] Hassanein K, Head M (2007). Manipulating perceived social presence through the web interface and its impact on attitude towards online shopping. International Journal of Human-Computer Studies.

[CR12] Jang, S. (2022). *A study on designer’s haptic perception of fashion fabrics in digital environment* [Doctoral thesis, Seoul National University]. SNU Open Repository and Archive. https://dcollection.snu.ac.kr/common/orgView/000000170316

[CR13] Jang S, Ha J (2021). The influence of tactile information on the human evaluation of tactile properties. Fashion and Textiles.

[CR14] Kok A., de Jong H.Looren (1980). The effect of repetition of infrequent familiar and unfamiliar visual patterns on components of the event-related brain potential. Biological Psychology.

[CR15] Kahrimanovic M, Bergmann Tiest WM, Kappers AML (2011). Discrimination thresholds for haptic perception of volume, surface area, and weight. Attention, Perception, & Psychophysics.

[CR16] Musa ABH, Malengier B, Vasile S, Van Langenhove L (2019). A comprehensive approach for human hand evaluation of split or large set of fabrics. Textile Research Journal.

[CR17] Negash S, Ryan T, Igbaria M (2003). Quality and effectiveness in Web-based customer support systems. Information & Management.

[CR18] Oh K (2006). A study on multimodal interface for human-computer interaction (HCI) improvement—focusing on the element of the tactile in the haptic interface. Journal of Digital Design.

[CR19] Petreca, B., Baurley, S., & Bianchi-Berthouze, N. (2015). How do designers feel textiles? In: *Proceedings of international conference on affective computing and intelligent interaction* (pp. 982–987). IEEE. 10.1109/acii.2015.7344695

[CR20] Volino Pascal, Davy Pierre, Bonanni Ugo, Luible Christiane, Magnenat-Thalmann Nadia, Mäkinen Mailis, Meinander Harriet (2007). From measured physical parameters to the haptic feeling of fabric. The Visual Computer.

[CR21] Xiao B, Bi W, Jia X, Wei H, Adelson EH (2016). Can you see what you feel? Color and folding properties affect visual–tactile material discrimination of fabrics. Journal of Vision.

[CR22] Xue Z, Zeng X, Koehl L, Chen Y (2013). Extracting fabric hand information from visual representations of flared skirts. Textile Research Journal.

